# Brain Perfusion Scintigraphy in the Diagnostic Toolbox for the Confirmation of Brain Death: Practical Aspects and Examination Protocol

**DOI:** 10.3390/diagnostics15212734

**Published:** 2025-10-28

**Authors:** Albrecht Günther, Anne Gunkel, Christian Geis, Dirk Brämer, Martin Brauer, Claus Doerfel, Michael Fritzenwanger, Martin Freesmeyer, Thomas Winkens, Robert Drescher, Anke Werner

**Affiliations:** 1 Department of Neurology, Jena University Hospital, 07747 Jena, Germany; 2 Department of Anesthesiology and Intensive Care Medicine, Jena University Hospital, 07747 Jena, Germany; 3 Department of Pediatrics, Pediatric Intensive Care Medicine and Neonatology, Jena University Hospital, 07747 Jena, Germany; 4 Department of Internal Medicine, Jena University Hospital, 07747 Jena, Germany; 5 Department of Nuclear Medicine, Jena University Hospital, 07747 Jena, Germanyrobert.drescher@med.uni-jena.de (R.D.); anke.werner@med.uni-jena.de (A.W.)

**Keywords:** brain perfusion scintigraphy, ILBF, ancillary tests, organ donation

## Abstract

**Background:** In addition to clinical examinations, confirmatory investigations are frequently performed to determine brain death (BD). Among other perfusion tests, brain perfusion scintigraphy (BPS) has been shown to be a reliable tool for the detection of brain circulatory arrest, particularly in cases with inconclusive clinical status or potential pharmacological interactions. **Methods**: Evaluation for brain death included standardized clinical examinations by two experienced neuro-intensive medicine specialists, followed by ancillary brain perfusion tests. BPS with the lipophilic tracer ^99m^Tc-hexamethylpropyleneamine oxime (HMPAO) was performed according to a standardized protocol. Imaging results, additional confirmatory test results, as well as clinical parameters were prospectively recorded. **Results:** BPS was performed in 30 patients (18 male, 12 female; median age 55.5 years, range 0.1–79.8 years). Eight patients underwent decompressive craniectomy (DC) prior to BD evaluation, three patients were supported by veno-arterial extracorporeal membrane oxygenation (vaECMO), and one patient by a left ventricular assist device (LVAD). The median interval between the initial brain-damaging event and BPS was 4.0 days (range 1–18 days). BPS demonstrated brain perfusion cessation in all patients. A concomitant single-photon emission computed tomography (SPECT) was required in one patient. There were no technical failures requiring a re-examination. **Conclusions:** BPS is a feasible, safe, and technically robust confirmatory test in BD diagnosis. BPS yielded unambiguous results, particularly in cases with inconclusive results of other ancillary tests, in neonates, young children and patients after DC. It is applicable to patients supported by LVAD and vaECMO.

## 1. Introduction

The determination of brain death (BD), defined as the irreversible loss of brain function (ILBF), remains a critical process in intensive care medicine, with profound clinical, ethical, legal, and even religious implications [1,2,3]. While clinical examination is the gold standard for BD diagnosis, it is not always feasible or conclusive. In such conditions, ancillary tests are required to confirm the absence of cerebral function. These include examination of cerebral circulation, i.e., digital subtraction angiography (DSA), computed tomography angiography (CTA), transcranial color-coded duplex sonography (TCCS), and brain perfusion scintigraphy (BPS). Further tests assess neurophysiological activity, i.e., electroencephalogram (EEG) and evoked potentials (EP). Each of these tests has distinct limitations in clinical settings, such as substantial cranial structural damage or the presence of profound sedation [4].

Among the ancillary tests, BPS is not a new method. Using lipophilic radiopharmaceuticals, it was first introduced in the 1980s and has gained acceptance for its ability to visualize the cessation of intracerebral blood flow and perfusion [5,6,7]. Following intravenous administration, the technetium-99m-labeled hexamethylpropyleneamine oxime (^99m^Tc-HMPAO) crosses the blood–brain barrier and distributes within the brain tissue proportionally to regional cerebral blood flow. In addition to ancillary testing in BD, it can be applied to evaluate patients with cerebrovascular diseases, such as acute stroke or chronic ischemia, for the preoperative localization of epileptogenic foci, as well as assessing patients showing evidence of dementia [8,9].

At our tertiary care university hospital, which has a large transplantation center, BD-related BPS examinations have been performed regularly since 2020. This included the establishment of an institutional standard operating procedure (SOP), the continuous availability of radiopharmaceuticals, quality control protocols, and staff training in imaging acquisition and interpretation. This study provides a comprehensive evaluation of all BPS procedures performed to date at our center in the context of BD determination. Diagnostic performance and feasibility of BPS in clinical practice, including for challenging patient groups such as infants, patients on extracorporeal membrane oxygenation (ECMO), and individuals with decompressive craniectomy (DC), are evaluated. We describe technical parameters, image acquisition protocols, and interpretation criteria, and critically discuss the role of BPS in the context of current guidelines and alternative confirmatory methods.

## 2. Materials and Methods

### 2.1. Patient Characteristics

**Figure 1:**
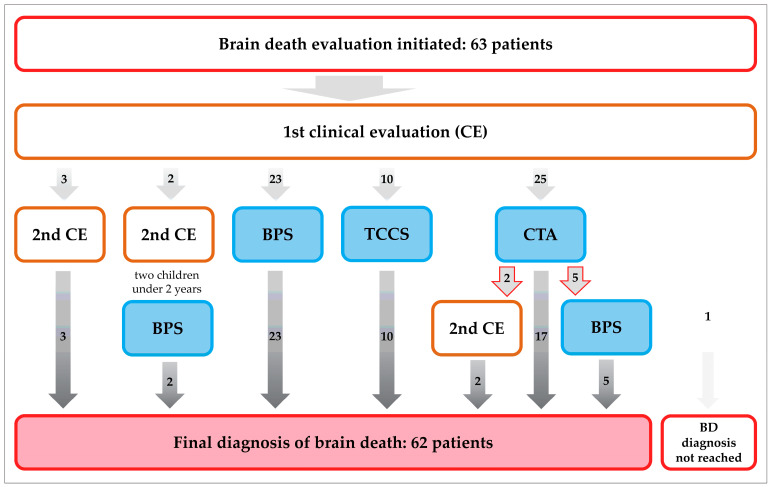
During the study period, 63 patients underwent formal brain death evaluation according to the German guidelines for BD determination [2]. In 60 patients, ancillary brain perfusion tests (BPS, CTA, and TCCS) were performed, of which seven CTA were inconclusive (red arrows). In children under two years of age, two clinical evaluations are mandatory. Brain death was confirmed in 62 patients. In one patient with a large ICH, CTA showed perfusion in parts of the brain, and the diagnosis of BD was not reached.

Between September 2020 and June 2025, 63 patients underwent standardized BD evaluation in our hospital (Figure 1). In 60 patients, ancillary perfusion tests (BPS, TCCS, or CTA) were performed after the first clinical examination or, in the case of infants below the age of two years, after the second clinical examination, according to the German guidelines [2]. Thirty patients (18 male, 12 female; median age 55.5 years, range 0.1–79.8 years, IQR 47.6 years) who underwent BPS were consecutively included in this evaluation (Figure 1).

The initial events causing brain damage were classified as traumatic, hemorrhagic stroke, ischemic stroke, hypoxic–ischemic encephalopathy, infectious, and other causes. Patient age and sex, imaging findings, history of DC, ECMO/LVAD (if applicable), and the use of additional confirmatory tests were recorded. Intervals between the initial brain-damaging event, DC (if applicable), and BPS were calculated.

### 2.2. Scintigraphic Method

BPS examinations were performed according to the German Medical Association (Bundesärztekammer) guidelines regarding technical, interpretational, and quality control aspects [2]. Guidelines of the German Society of Nuclear Medicine (DGN), European Association of Nuclear Medicine (EANM), and the Society of Nuclear Medicine and Molecular Imaging (SNMMI) for BPS were considered [10,11]. A summary of the procedure is provided in the Supplementary Materials.

HMPAO is currently available in the following commercial formulations: Ceretec (GE Healthcare, Oslo, Norway), which is usable for up to 6 h after labeling with ^99m^Tc; Medi-Exametazim (Medi-Radiopharma, Érd, Hungary), usable for one hour after labeling; and Stabilised Brain-SPECT (Medi-Radiopharma, Érd, Hungary), usable for up to 6 h after labeling. The latter is not approved for use in Germany but may be used in the absence of an alternative.

The ^99m^Tc-HMPAO kits (Ceretec GE Healthcare, Oslo, Norway) have a shelf life of 6 months in a refrigerator (max. 8 °C). Radiolabeling was performed according to the manufacturer’s instructions by transferring pertechnetate (TcO_4_^−^) solution into a vial containing the dry precursor substance, followed by incubation at room temperature for 5 min. The resulting product was usable for up to one hour. By adding a cobalt-containing stabilization agent, the shelf-life of the radiolabeled compound could be extended to six hours [8,9]. Radiopharmaceutical purity was assessed using two different thin-layer chromatography (TLC) methods using ITLC-SA chromatography paper to detect free pertechnetate (eluent: isotonic saline solution) and technetium colloid or secondary complexes (eluent: methyl ethyl ketone), respectively. The radiochemical purity should be above 90%.

Imaging was performed on a gamma camera with SPECT/CT (Symbia Intevo Bold, Siemens Healthineers, Erlangen, Germany) or SPECT (Symbia 2, Siemens Healthineers, Erlangen, Germany) capabilities. Patients were positioned supine with the head immobilized. German Society of Nuclear Medicine (DGN) guidelines and kit manufacturer’s recommendations suggest doses of 400–740 MBq and between 555 and 1110 MBq ^99m^Tc-HMPAO for BPS, respectively [8,9,12]. Our standard dose is 700–740 MBq ^99m^Tc-HMPAO for adults, and bodyweight- and age-adapted doses for children, based on the EANM dosage card [13]. The tracer was injected in a 10 mL saline bolus, followed by a 20 mL saline flush through a peripheral intravenous cannula. Port systems or central venous catheters were avoided to prevent image distortions arising from high activity concentration in the subclavian and superior caval veins.

Dynamic acquisition of the head and neck (anterior view, low energy high resolution (LEHR) collimator, matrix 64 × 64, zoom 1.0) was started simultaneously with the injection and proceeded for 2 min (12 frames, 2 s per frame, followed by 12 frames, 8 s per frame) to confirm tracer flow to the carotid arteries. Biplanar static acquisitions of the head were acquired, starting 30 min after tracer injection (anterior–posterior and lateral views, matrix 128 × 128, zoom 1.0, 5 min or 500,000 counts per view). In ambiguous cases, but also to assess its complementary diagnostic value in BD determination, an additional SPECT of the head (32 angles, 20 s per angle) was performed. As a final quality control step, an anterior planar image of the abdomen was acquired. Prominent distribution in highly perfused organs, such as the liver and spleen, confirms correct application and systemic circulation of the tracer.

A possible workflow sheet for routine use is provided in the Supplementary Materials.

### 2.3. Image Evaluation

The images were analyzed by nuclear medicine specialists familiar with the technique. The absence of scintigraphic tracer flow into the brain (on the early, dynamic images), combined with the absence of tracer uptake in the brain (on the late, static images or on SPECT), defined the loss of brain perfusion. The classifications “no evidence of brain perfusion” and “evidence of brain perfusion” without further gradation were used in the final reports.

For study purposes, the semi-quantitative delayed uptake index (DUI) was calculated as the ratio of ^99m^Tc-HMPAO uptake in the brain to that in the facial region, measured on the late planar images [14]. Accordingly, measurements were performed using a syngo.via workstation (MM Oncology module, Siemens Healthineers, Erlangen, Germany). Regions of interest (ROIs) were manually delineated on lateral views. One ROI was positioned in the facial area with attention to avoid hyperperfused salivary glands. The other ROI was placed over the brain, with attention to exclude the calvarium. The mean counts within each ROI on the left and right facial regions, as well as the mean counts within each ROI on the left and right brain regions, were calculated and subsequently averaged.

### 2.4. Statistical Evaluation

Statistical evaluation was carried out using Excel and Access (Microsoft, Redmond, WA, USA) and SPSS 29.0 for Windows (IBM Corp., Armonk, NY, USA). For descriptive statistics, absolute numbers with mean, standard deviation, median, interquartile ranges, and percentages are reported.

## 3. Results

### 3.1. Patient Characteristics

Twenty-five patients received a BPS as the primary ancillary test, and five patients as the secondary ancillary test after an inconclusive CTA (Figure 1, Table 1). The most common initial events were hemorrhagic (33.3%), followed by hypoxic (23.3%; mostly hypoxic–ischemic brain damage following cardiac arrest), traumatic (20%), and ischemic events (16.7%). Eight patients (26.7%) underwent DC within 48 h after the initial event. Three patients were examined while on veno-arterial extracorporeal membrane oxygenation (vaECMO). One patient had recently undergone implantation of a left ventricular assist device (LVAD).

### 3.2. Brain Perfusion Scintigraphy

All BPS examinations were technically successful. No scanner-, tracer-, or injection-related complications occurred. BPS was conducted within a range of 24 h to 18 days (median 4.0 days, IQR 8.8 days) after the initial brain-damaging event.

**Figure 2:**
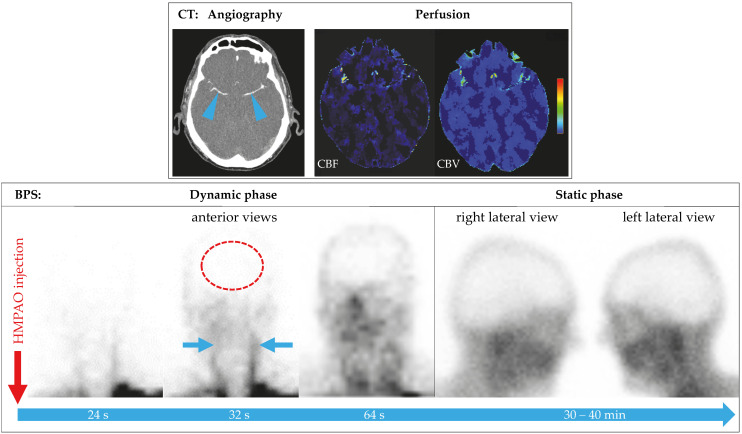
Patient no. 5 underwent brain death evaluation seven days after a cardiac arrest and prolonged CPR. (**Top row**)The CTA was considered inconclusive because the contrast medium was reaching both M2 middle cerebral arteries (arrowheads). CT perfusion showed loss of perfusion, but it is not an accepted ancillary perfusion test according to German guidelines [2]. **Bottom row**: The BPS of the same patient confirmed complete loss of brain perfusion. Planar dynamic images show tracer inflow into the common carotid arteries (arrows), confirming intra-arterial ^99m^Tc-HMPAO distribution. No tracer inflow into the brain (red oval), and no parenchymal tracer accumulation on the late static images is seen.

**Figure 3:**
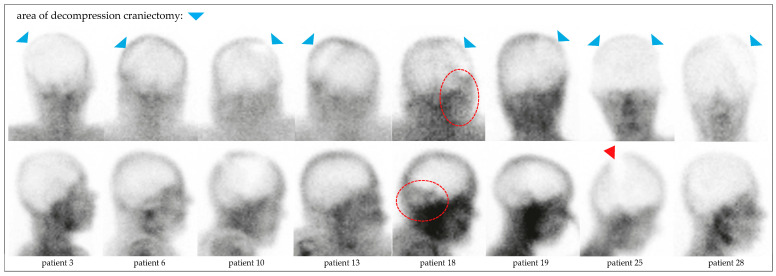
Planar scintigraphic images of all patients who underwent DC, posterior (**upper row**) and right lateral (**lower row**) views. The craniectomy defects (blue arrowheads) were best visualized from the posterior view. In patient no. 18, activity was seen projecting onto the cerebellum (red circle), which was further evaluated by SPECT/CT (see also Figure 4). In patient no. 25, the stripe of low uptake on the lateral view (red arrowhead) correlated with the long postoperative scalp suture following bilateral DC.

**Figure 4:**
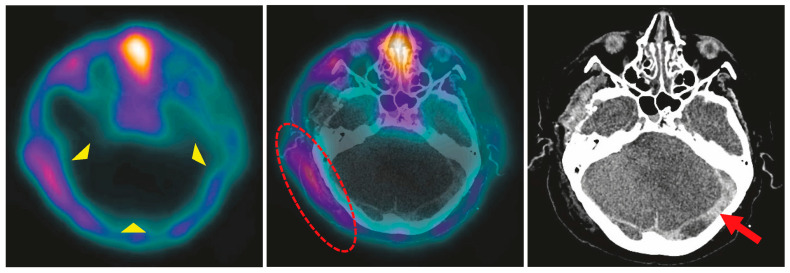
SPECT (**left**), SPECT/CT fusion (**middle**), and CT images (**right**) of patient no. 18. The axial SPECT reconstruction confirms the loss of perfusion in the cerebellum (yellow arrowheads). The tracer activity seen on the lateral planar image of this patient corresponds to hyperperfused, extracranial swollen tissue (red oval). Subarachnoid hemorrhage is present (red arrow).

In all patients, BPS showed complete loss of brain perfusion. In 29 patients (96.7%), the diagnosis could be established based on dynamic and static planar images (Figure 2). In a 71-year-old male with ischemic stroke, secondary ICH, and intermittent DC, residual infratentorial perfusion was suspected on planar images (patient no. 18, Figure 3). The additional SPECT/CT scan revealed asymmetric soft tissue abnormalities around the skull base and neck, confirming the complete loss of brain perfusion (Figure 4). SPECT was performed in an additional eleven patients at the discretion of the respective nuclear medicine physicians, confirming the loss of perfusion seen on planar images.

Overall, prior DC did not impair the reliability of BPS. In patients on veno-arterial ECMO (no. 5, 16, 29) and in the patient with an LVAD (no. 26), BPS yielded unequivocal results.

### 3.3. Semiquantitative Evaluation

DUI values were calculated for all cases and showed a median of 0.218 (range 0.080–0.328, IQR 0.083; Table 1).

## 4. Discussion

In the present study, we report on our experience using BPS to prove brain perfusion cessation in BD patients. Several uncommon clinical conditions in our cohort deserve attention and add knowledge to the previous BPS series, which did not include patients supported by vaECMO or LVAD in the context of BD.

From a nuclear medicine perspective, the imaging acquisition component of BPS, comprising planar dynamic and static scans, is straightforward and less complex than other examinations, such as myocardial perfusion or renal function scintigraphy. The more important late static acquisitions can be repeated if necessary, up to two hours after ^99m^Tc-HMPAO injection [11]. The tracer can be administered even through a small vein, provided it is not paravenous. The added complexity in BPS lies in the laboratory procedures. While these are generally simple due to kit-based tracer preparation, they require adequately trained staff to ensure the quality of the radiopharmaceutical. In an existing nuclear medicine department, the complexity and costs of establishing BPS examinations in clinical routine are relatively low.

Quantitative analysis using the delayed uptake index (DUI) was implemented in our study to strengthen the objectivity and standardize assessment within the evaluation, providing an objective metric to support visual interpretation of perfusion patterns; however, its clinical utility remains limited without widely accepted thresholds [14]. The values measured in our patients were higher than those described by Mrhac et al. in BD patients, probably due to the more sensitive scanning technology used today compared to 1995, but still markedly lower than in their non-BD group. Further research regarding SPECT/CT quantification is needed. Finally, although Dickson et al. pointed out the lack of standardization in quantitative SPECT calibration across institutions, our protocol prioritized visual assessment in accordance with the guidelines and was supplemented by quantitative data for research purposes [15]. The additional value of PET radiopharmaceuticals, such as ^18^F-fluorodeoxyglucose, for BPS has yet to be evaluated [16,17]. The radiation exposure associated with BPS does not constitute a significant limitation for its clinical use. Due to the short physical half-life of technetium-99m (6 h) and the low administered activity, absorbed radiation doses to organs remain minimal, and no significant radiation exposure to surgical or medical personnel is observed [10].

SPECT or SPECT/CT acquisition should be performed in equivocal planar studies to enhance anatomical resolution, particularly to differentiate residual brain perfusion from overlying, perfused extracranial tissues (e.g., scalp, parotid glands, facial tissues) as visualized in one of our patients (Figure 4). Even though it is not mandatory according to guidelines, the easier evaluation of tomographic images is an advantage in BD patients to avoid ambiguity. Previous studies have shown that BPS with brain-specific agents, such as ^99m^Tc-HMPAO, allows sufficient visualization of the brainstem and posterior fossa [18,19]. SPECT(/CT), in addition to planar images, is advisable in uncertain cases.

### 4.1. BPS in the Context of Guidelines

Brain perfusion scintigraphy is one of the ancillary tests endorsed by international and national guidelines for brain death determination. The German Federal Medical Association considers BPS a valid confirmatory test in all age groups and brain injury entities, particularly when clinical examination is compromised or confounding factors, such as impossible apnea testing or residual sedation, cannot be excluded [2]. Zukier et al. emphasized the practical advantages of radionuclide perfusion studies: high diagnostic reliability, ease of use, and minimal susceptibility to pharmacologic or metabolic confounders. Importantly, lipophilic radiopharmaceuticals, such as ^99m^Tc-HMPAO, enable parenchymal imaging by crossing the blood–brain barrier and becoming trapped in viable neural cells, making them superior to flow-only, hydrophilic agents [20]. According to the American Academy of Neurology Consensus guidelines, the World Brain Death Project, and the German guidelines, if scintigraphic techniques are employed, they should use lipophilic radiopharmaceuticals and, if possible, include tomographic imaging (SPECT) to increase spatial resolution and diagnostic confidence [3].

Sinha et al. reaffirmed that the absence of both tracer flow in the brain supplying vessels and uptake of lipophilic radiotracers in the brain, in the appropriate clinical context, is consistent with brain death [21]. These recommendations are echoed by Bohatyrowicz et al. in the Polish guidelines, highlighting the interpretative clarity BPS provides to physicians and family members alike: non-perfused brain tissue is intuitively understood as non-viable, even without specialized knowledge [22].

### 4.2. BPS in Infants and Children

One of the most important applications of BPS is in pediatrics, where clinical confirmation of brain death is particularly challenging. In infants, open fontanelles and immature cerebral structures often pose significant challenges to conventional imaging modalities, resulting in suboptimal image quality or inconclusive findings. McKinnon et al. demonstrated that radionuclide scintigraphy using ^99m^Tc-HMPAO, regardless of SPECT availability, e [23]. An important nuance in interpretation involves uptake in dural venous sinuses, observed in some pediatric cases [24]. This uptake does not contradict a diagnosis of BD if there is no tracer retention in the brain parenchyma. Our institutional practice followed the EANM pediatric dosage recommendations, enabling accurate and safe tracer administration across all pediatric age groups [13].

### 4.3. BPS in Hemicraniectomy

Decompressive hemicraniectomy poses a specific challenge for most perfusion-based confirmatory tests, especially CTA, due to altered intracranial pressure dynamics and disrupted vascular flow patterns [25]. Evaluation of the planar images can be difficult due to perfused overlying tissues. Known findings include hyperperfusion of the paranasal tissues (“hot nose” sign on anterior–posterior views), as well as residual flow in the sagittal sinus arising from external carotid artery and meningeal artery branches. Asymmetric perfusion of the scalp can be misleading in patients who have undergone decompressive craniectomy, which is mostly performed unilaterally. In one of our patients, prior surgery had resulted in asymmetric overlying soft tissues, mimicking cerebellar perfusion on planar images. This finding was clarified using SPECT/CT (Figure 2 and Figure 3). As suggested by Zukier et al., the addition of SPECT in these cases helps distinguish between extracranial uptake (e.g., scalp, parotid glands) and potential residual brain perfusion [20]. BPS thus overcomes one of the major pitfalls of angiographic modalities in this subgroup. In summary, our experience confirms prior findings that BPS remains clearly interpretable even in large skull defects [26].

### 4.4. BPS in Patients on vaECMO or LVAD

Confirming brain death in patients on ECMO presents unique difficulties. Traditional flow-based methods often yield unreliable results due to altered hemodynamics [27]. As demonstrated by Günther et al. [28] into brain perfusion and circulation conditions in clinically brain-dead patients. Brain perfusion investigations, especially using BPS, in LVAD patients in the context of BD have, to the best of our knowledge, not yet been published.

### 4.5. BPS Compared with Other Ancillary Tests

Munari et al. found a 100% concordance between digital subtraction angiography (DSA) and BPS in brain-dead patients, with perfusion scintigraphy offering superior visualization of the posterior fossa and brainstem regions where DSA or CTA can be limited [7]. Furthermore, BPS does not require the administration of iodine contrast medium or adjustment of ventilator settings, reducing procedural risk, especially in hemodynamically unstable patients [29]. Contrast-related opacification of intracranial vessels may be considered evidence of intracranial circulation, although it may merely reflect a transient increase in arterial blood pressure exceeding intracranial pressure. Furthermore, false-positive results have been reported for CTA, showing no residual flow, whereas transcranial Doppler ultrasound demonstrated preserved cerebral blood flow [3,30].

Compared to TCCS, BPS is less operator-dependent and not affected by acoustic window limitations. Its functional nature provides direct evidence of parenchymal viability, whereas TCCS and CTA rely on demonstrating absent or stagnant flow as an indirect indicator of brain circulatory arrest in clinically brain-dead patients.

CTA presents greater technical challenges during image acquisition. Precise bolus injection, timing, and tracking are critical, and even minimal patient movement can significantly impair image quality. Furthermore, contrast medium injection cannot be repeated immediately in case of a technical problem, and may also be harmful for patients with renal insufficiency. A key advantage of CTA is the immediate availability of contrast agents.

In contrast, BPS evaluates tracer uptake at the tissue level, not just vascular flow. From an imaging standpoint, the focus is not restricted to evaluating small arterial segments but extends to the entire brain parenchyma, resulting in a straightforward “snapshot” of regional cerebral blood flow at the moment of injection [31].

In our cohort, BPS provided clarity in cases where CTA results were non-confirmatory or ambiguous, supporting its role as a problem-solving tool in multimodal BD diagnostics.

Of the 63 patients evaluated for BD in the study period, only 10 patients underwent TCCS as the ancillary perfusion test. This low number can be attributed to the fact that TCCS is also performed in many patients prior to the formal BD determination procedure, thereby allowing for the determination of ultrasound feasibility, i.e., the presence of a transcranial bone window. Furthermore, non-operator-dependent methods, such as CTA and BPS, are preferred.

Despite its strengths, BPS has limitations. Interpretation may be compromised by extracerebral tracer uptake or suboptimal injection technique. Careful quality control, including abdominal imaging to confirm tracer circulation, is therefore essential. The main limitations of this study are its small sample size and its observational nature, which precluded a systematic comparison of BPS accuracy with other ancillary tests.

## 5. Conclusions

This study demonstrates the clinical feasibility, diagnostic accuracy, and thus the relevance of BPS in confirming BD, and confirms its robustness in routine use, particularly in cases with incomplete investigability or inconclusive clinical findings. Technically, BPS is also a relatively simple nuclear medicine examination. In our experience with such patients, such as infants, patients on vaECMO, or LVAD, the latter, to our knowledge, has not previously been reported for brain perfusion assessment using BPS in BD patients. A previous DC did not complicate interpretation. However, SPECT acquisition appears advisable to reliably exclude false interpretations caused by overlying extracerebral tissue.

The practical overview and protocol chart for BPS, provided in two languages (English and German), serve as supplements to facilitate the broader adoption of the method and should be considered practical recommendations.

In conclusion, further implementation of BPS in the diagnostic algorithm for BD patients may decrease the number of inconclusive and, thus, potentially false negative results without a risk of false positive results when strictly following the established clinical and ancillary test protocols.

## Figures and Tables

**Table 1:** Clinical characteristics and brain perfusion scintigraphy results.

Patient *	Age	Initial Event	Initial Imaging Findings	DC	Brain Perfusion Scintigraphy			DUI
	Years			Days After Event	Days After Event	Arterial Blood Pressure (mm Hg)	Perfusion Detected	
**Traumatic (** ***n*** ** = 6)**								
1	2.1	NAHI	SDH, SAH, brain edema	-	2	115/73	no	0.083
3	14.7	traffic accident	EDH, SDH, contusion, axonal injuries, brain edema, spinal fractures	0	2	130/74	no	0.194
4	0.1	NAHI	axonal injuries, brain edema, SDH, SAH	-	4	94/51	no	0.176
27 **	62.7	gunshot injury	ICH, SAH	-	1	123/79	no	0.304
28	15.0	traffic accident	ICH, SDH, brain edema	1	1	129/85	no	0.173
30	33.1	cervical fractures, VA dissection	brain edema	-	1	125/71	no	0.153
**Hemorrhagic (** ***n*** ** = 10)**								
2	56.9	spontaneous ICH	ICH, brain edema	-	2	125/65	no	0.225
6	55.1	aneurysm rupture	SAH, brain edema, brain ischemia	0	14	90/60	no	0.226
7 **	62.1	aneurysm rupture	SAH, brain edema, brain ischemia	-	10	95/45	no	0.263
10	42.3	aneurysm rupture	SAH, brain edema	1	15	143/78	no	0.213
11 **	55.2	aneurysm rupture	SAH, ICH, brain edema	-	13	120/77	no	0.258
12	70.6	basilar artery rupture	SAH	-	2	146/62	no	0.187
13	48.1	spontaneous ICH	ICH, brain edema, brain ischemia	1	18	123/56	no	0.298
17	79.8	spontaneous ICH	ICH, brain edema	-	3	164/85	no	0.253
19	34.8	aneurysm rupture	SAH	1	11	122/66	no	0.172
26	62.2	ICH after ischemia	brain ischemia, ICH	-	5	123/78 (LVAD)	no	0.239
**Hypoxic (** ***n*** ** = 7)**								
5 **	62.2	cardiac arrest, CPR	brain edema	-	7	106/77 (vaECMO)	no	0.158
8 **	39.3	cardiac arrest, CPR	brain edema, ischemia, SAH	-	16	154/90	no	0.271
14	55.8	cardiac arrest, CPR	SAH, brain edema	-	8	138/75	no	0.295
16	58.4	cardiac arrest, CPR	anoxic brain injury, brain edema	-	4	101/64 (vaECMO)	no	0.198
21	14.0	smoke inhalation	brain edema	-	1	115/63	no	0.203
23	1.8	non-diphtheric croup, cardiac arrest, CPR	brain edema	-	2	95/51	no	0.083
29	72.6	cardiac arrest	brain edema	-	3	121/75 (vaECMO)	no	0.286
**Ischemic stroke (** ***n*** ** = 5)**								
9	79.6	brain ischemia	ischemia, brain edema	-	5	188/80	no	0.234
15	62.0	brain ischemia	ischemia, SAH	-	2	110/70	no	0.328
18	71.3	brain ischemia	ischemia, ICH	0	14	148/68	no	0.205
20	64.8	brain ischemia	ischemia	-	4	135/85	no	0.222
22	69.0	brain ischemia	ischemia, brain edema	-	3	115/62	no	0.196
**Infectious (** ***n*** ** = 2)**								
24	13.9	encephalitis	brain edema	-	11	124/84	no	0.326
25	13.1	pneumococcal meningitis	brain edema	1	7	118/75	no	0.165

CPR, cardiopulmonary resuscitation; DC, decompressive craniectomy; DUI, delayed uptake index; EDH/SDH/SAH/ICH, epidural/subdural/subarachnoid/intracerebral hemorrhage; NAHI, non-accidental head injury; LVAD, left ventricular assist device; vaECMO, veno-arterial extracorporeal membrane oxygenation; *, consecutive numbering; **, inconclusive prior CTA.

## Data Availability

Data are available upon request due to privacy restrictions and ethical considerations.

## Supplementary Materials

The following supporting information can be downloaded at: https://www.mdpi.com/article/10.3390/diagnostics15212734/s1, Documents: Supplement_BPS Protocol overview and chart proposal_English.pdf; Supplement_BPS Protocol overview and chart proposal_German.pdf.

## Abbreviations

The following abbreviations are used in this manuscript:

BD	brain death
BPS	brain perfusion scintigraphy
CPR	cardiopulmonary resuscitation
CTA	computed tomography angiography
DC	decompressive craniectomy
DGN	Deutsche Gesellschaft für Neurologie (German Neurological Society)
DGN	Deutsche Gesellschaft für Nuklearmedizin (German Society of Nuclear Medicine)
DSA	digital subtraction angiography
DUI	delayed uptake index
EANM	European Association of Nuclear Medicine
EDH/SDH/SAH/ICH	epidural/subdural/subarachnoid/intracerebral hemorrhage
EEG	electroencephalogram
EP	evoked potentials
HMPAO	hexamethylpropyleneamine oxime
ILBF	irreversible loss of brain function
IQR	interquartile range
LEHR	low energy high resolution
LVAD	left ventricular assist devices
NAHI	non-accidental head injury
e	regions of interest
SNMMI	Society of Nuclear Medicine and Molecular Imaging
SOP	standard operating procedure
SPECT	single-photon emission computed tomography
e	thin-layer chromatography
vaECMO	veno-arterial extracorporeal membrane oxygenation
