# Supplementary material for: Brain Perfusion Scintigraphy in the Diagnostic Toolbox for the Confirmation of Brain Death: Practical Aspects and Examination Protocol
Source: Diagnostics (Basel). 2025 Oct 28;15(21):2734. doi: 10.3390/diagnostics15212734 (PMC12608010; doi:10.3390/diagnostics15212734)
Supplement: Supplementary file 1 [file diagnostics-15-02734-s001.zip › Supplement_BPS Protocol overview and chart proposal_English.pdf]

Brain Perfusion Scintigraphy with ^{99m}Tc-HMPAO for the diagnosis of brain death

Radiopharmaceutical preparation

- Possible kit: Ceretec™, GE Healthcare, Oslo, Norway
- Shelf life of 6 months in a refrigerator (max. 8°C)
- Radiolabeling takes approx. 45 min, main steps:
    - transfer of pertechnetate (TcO_4^-) solution into a vial containing the dry precursor substance
    - incubation at room temperature for 5 minutes
    - radiopharmaceutical purity tests, should be above 90%: tested with two thin-layer chromatography (TLC) methods, using ITLC-SA chromatography paper to detect free pertechnetate (eluent: isotonic saline solution) and technetium colloid or secondary complexes (eluent: methyl ethyl ketone)
- Product is usable for 1 hour (with cobalt-containing stabilization agent: 6 hours)

Patient and gamma camera setup

- Low energy high resolution (LEHR) collimator
- Patient in supine position, head fixated
- Check for respiration tubes and cables and external devices prior to table movement

Tracer dosage and injection

- Dose for administration:
    - 700-740 MBq ^{99m}Tc-HMPAO for adults,
    - bodyweight- and age-adapted doses for children (see EANM Dosage Card)
- Radiochemical purity OK, other radiopharmaceutical issues?
- Tracer injection in a 5-10 ml saline bolus, followed by a 20 ml saline flush through a peripheral intravenous cannula
- Injection into port systems or central venous catheters may cause image distortions arising from high activity concentration in the subclavian and superior caval veins

Image acquisition

- Dynamic acquisition of the head and neck (adjust zoom depending on patient height; anterior view only, matrix 64 x 64, 12 frames, 2 s per frame, followed by 12 frames, 8 s per frame) started simultaneously with the injection
- Static acquisitions of the head acquired from 30 min after tracer injection (2 detectors, anterior-posterior and lateral views, matrix 128 x 128, 5 min or 500000 counts per view)
- Additional SPECT or SPECT/CT of the head (32 angles, 20 s per angle) at the discretion of the nuclear medicine specialist
- Planar image of the abdomen (anterior view only, 5 min or 500000 counts)

Image evaluation

- By nuclear medicine specialists familiar with the technique
- Dynamic planar images of head/neck:
    - Scintigraphic tracer flow into the cervical arteries? (must be visible to confirm correct intravenous injection)
    - Scintigraphic tracer flow into the brain?
- Static planar images of head/neck:
    - Tracer uptake into the brain (supra/infratentorial)?
    - if unsure or overlying perfused tissue: add SPECT or SPECT/CT
- Planar abdominal images: Normal tracer distribution in abdominal organs (moderate in liver and spleen, low in stomach, low in connecting tissue)?

Report

- Recommended wording: „no evidence of brain perfusion“ or „evidence of brain perfusion“
- Do not use: „brain death“ or „irreversible loss of brain function“

Patient data

Referrer:

Ward:

Phone:

Clinical indication:

Notes:

To be completed by the Department of Nuclear Medicine

Quality control in vitro:

Radiochemical purity (free pertechnetate): ________%

Signature Radiopharmacy

Radiochemical purity (colloid, sec. complexes): ________%

Radiochemical purity (total): ________ %

Signature NM Specialist

Batch number:

Usable until:

QC protocol available: ☐

Application:

arterial BP at time of injection:

Injection site:

Injection time:

Syringe full	Syringe empty

Note (paravenous etc.):

Signature NM Specialist

Acquisition

Start times

1. dynamic acquisition __ : __
2. static acquisition (RVL/LDR, right/left lateral) __ : __
3. QC in vivo (chest/abdomen static) __ : __
4. SPECT (if necessary) __ : __

SPECT not performed because:

____

End of examination: __ : __

Signature technician
