# Supplementary material for: Brain Perfusion Scintigraphy in the Diagnostic Toolbox for the Confirmation of Brain Death: Practical Aspects and Examination Protocol
Source: Diagnostics (Basel). 2025 Oct 28;15(21):2734. doi: 10.3390/diagnostics15212734 (PMC12608010; doi:10.3390/diagnostics15212734)
Supplement: Supplementary file 1 [file diagnostics-15-02734-s001.zip › Supplement_BPS Protocol overview and chart proposal_German.pdf]

Hirnperfusionsszintigraphie mit ^{99m}Tc-HMPAO zur Diagnostik des Hirntods

Radiopharmazeutische Herstellung

- mögliches Kit: Ceretec™, GE Healthcare, Oslo, Norwegen
- Haltbarkeit: 6 Monate im Kühlschrank (max. 8°C)
- Radiomarkierung dauert ca. 45 Minuten, Hauptschritte:
  - Überführen der Pertechnetat (TcO_4^-)-Lösung in ein Fläschchen mit der trockenen Vorstufe
  - Inkubation bei Raumtemperatur für 5 Minuten
  - Prüfung der radiopharmazeutischen Reinheit, sollte über 90 % betragen: Test mit zwei Dünnschichtchromatographie-Methoden (TLC) unter Verwendung von ITLC-SA-Chromatographiepapier zum Nachweis von freiem Pertechnetat (Elutionsmittel: isotonische NaCl-Lösung) und Technetium-Kolloid oder Sekundärkomplexen (Elutionsmittel: Methylethylketon)
- Verwendbarkeit des Produkts: 1 Stunde (mit kobalthaltigem Stabilisator: 6 Stunden)

Patientenvorbereitung und Gammakameraeinstellung

- Kollimator: Low Energy High Resolution (LEHR)
- Lagerung: Patient in Rückenlage, Kopf fixiert
- Überprüfung auf Beatmungsschläuche, Kabel und externe Geräte vor Tischbewegung

Traceraktivität und Injektion

- Vorbereitung der Aktivität:
  - 700–740 MBq 99mTc-HMPAO für Erwachsene
  - Körpergewicht- und altersangepasste Dosierung für Kinder (siehe EANM Dose Card)
- Radiochemische Reinheit in Ordnung, andere radiopharmazeutische Probleme?
- Injektion über periphere Venenkanüle, Tracer in 5–10 ml NaCl-Lösung, anschließend Spülung mit 20 ml NaCl-Lösung
- die Injektion über Portsysteme oder zentrale Venenkatheter kann Bildartefakte durch hohe Aktivitätskonzentrationen in Vena subclavia und Vena cava superior verursachen

Bildakquisition

- dynamische Akquisition von Kopf und Hals (Zoom je nach Patientengröße anpassen; nur anteriore Ansicht, Matrix 64 x 64, 12 Bilder à 2 s, gefolgt von 12 Bildern à 8 s), Start gleichzeitig mit der Injektion
- statische Aufnahmen des Kopfes ab 30 Minuten nach Tracerinjektion (2 Detektoren, anterior-posteriore und laterale Ansichten, Matrix 128 x 128, 5 Minuten oder 500.000 Counts pro Ansicht)
- zusätzliche SPECT oder SPECT/CT des Kopfes (32 Winkel, 20 s pro Winkel) nach Ermessen des Nuklearmediziners
- planare Aufnahme des Abdomens (nur anteriore Ansicht, 5 Minuten oder 500.000 Counts)

Bildauswertung

- Durchführung durch mit der Methode vertrautem Facharzt für Nuklearmedizin
- dynamische planare Bilder von Kopf/Hals:
  - Tracereinstrom in die zervikalen Arterien? (muss sichtbar sein zur Bestätigung korrekter intravenöser Injektion)
  - Tracereinstrom ins Gehirn?
- Statische planare Bilder von Kopf/Hals:
  - Traceruptake ins Gehirn (supratentoriell/infratentoriell)?
  - bei Unsicherheiten oder überlagerndem perfundiertem Gewebe: SPECT oder SPECT/CT ergänzen
- planare Aufnahme des Abdomens: Normale Tracerverteilung in den abdominellen Organen (moderat in Leber und Milz, gering im Magen, gering im Bindegewebe)?

Befund

- empfohlene Formulierungen: „Kein Hinweis auf Hirnperfusion“ bzw. „Hinweis auf Hirnperfusion“
- nicht verwenden: „Hirntod“ oder „Irreversibler Funktionsausfall des Gehirns“

Patientenetikett

Klinik:

Station:

Telefon:

Fragestellung:

Anmerkungen:

Nur von der Klinik für Nuklearmedizin auszufüllen:

Qualitätskontrolle in vitro: Radiochem. Reinheit (freies Pertechnetat): _______% ____________________

Unterschrift Radiopharmazie

Radiochem. Reinheit (Kolloid, Sekundärkomplex): _______%

Radiochem. Reinheit (gesamt): _______% ____________________

Unterschrift Facharzt

Chargennummer:

verwendbar bis:

QK-Protokoll vorliegend: ☐

Applikation:

Injektionsort:

Injektionszeit:

Spritze voll

Spritze leer

RR zum Injektionszeitpunkt:

Bemerkung (para etc):

____

Unterschrift Facharzt

Akquisition

Startzeiten

1. dynamische Akquisition __ : __ Uhr
2. statische Akquisition (RVL/LDR, re./li. lat.) __ : __ Uhr
3. QK in vivo (Thorax/Abdomen statisch) __ : __ Uhr
4. SPECT (falls erforderlich) __ : __ Uhr

Kein SPECT durchgeführt weil: ____________________

Untersuchungsende: __ : __ Uhr

____

Unterschrift MTA
